# Supplementary material for: Bridging Brain and Cognition: A Multilayer Network Analysis of Brain Structural Covariance and General Intelligence in a Developmental Sample of Struggling Learners
Source: J Intell. 2021 Jun 15;9(2):32. doi: 10.3390/jintelligence9020032 (PMC8293355; doi:10.3390/jintelligence9020032)
Supplement: Supplementary file 1 [file jintelligence-09-00032-s001.zip › jintelligence-1108256-supplementary.pdf]

SUPPLEMENTARY MATERIAL

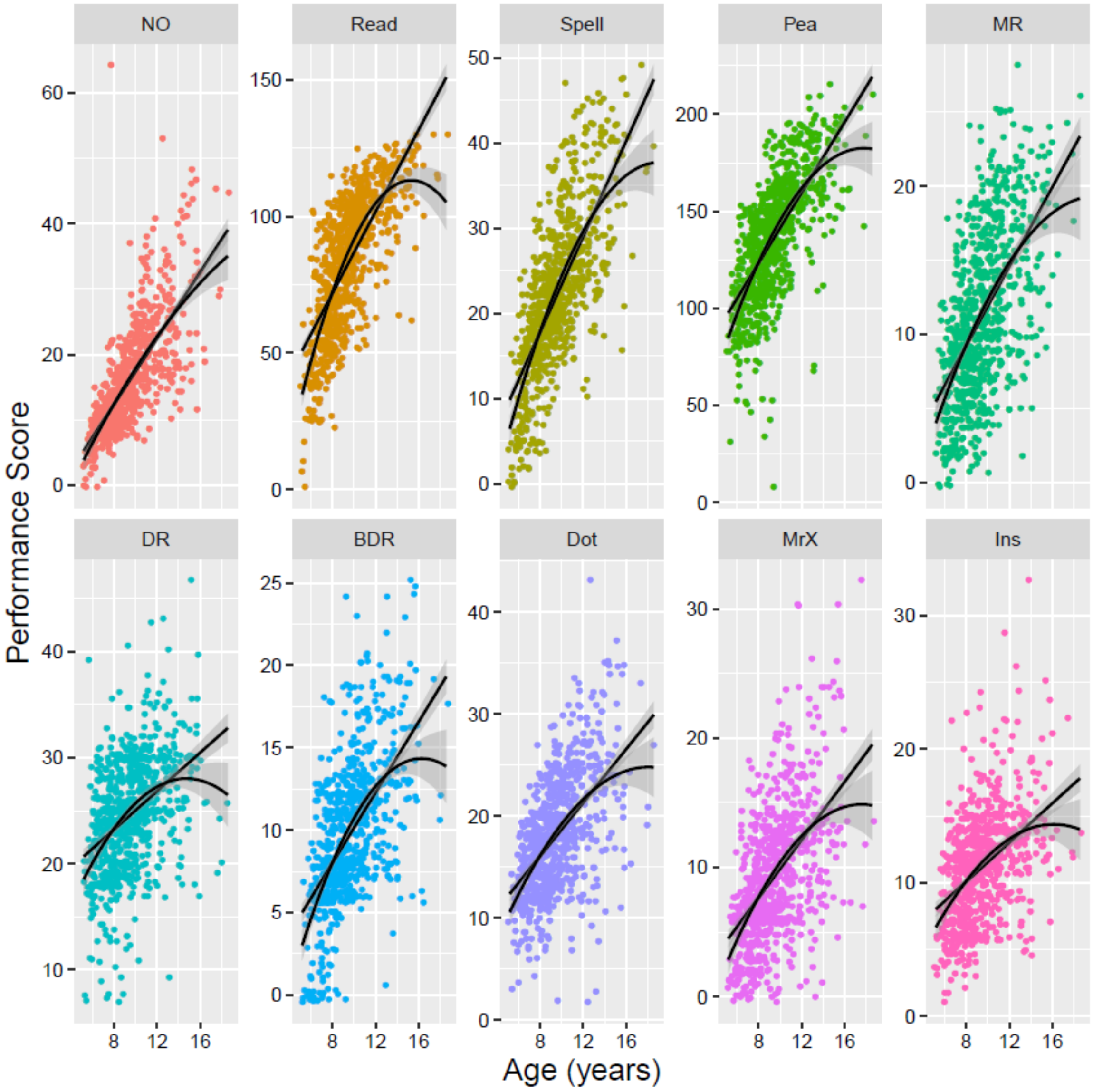

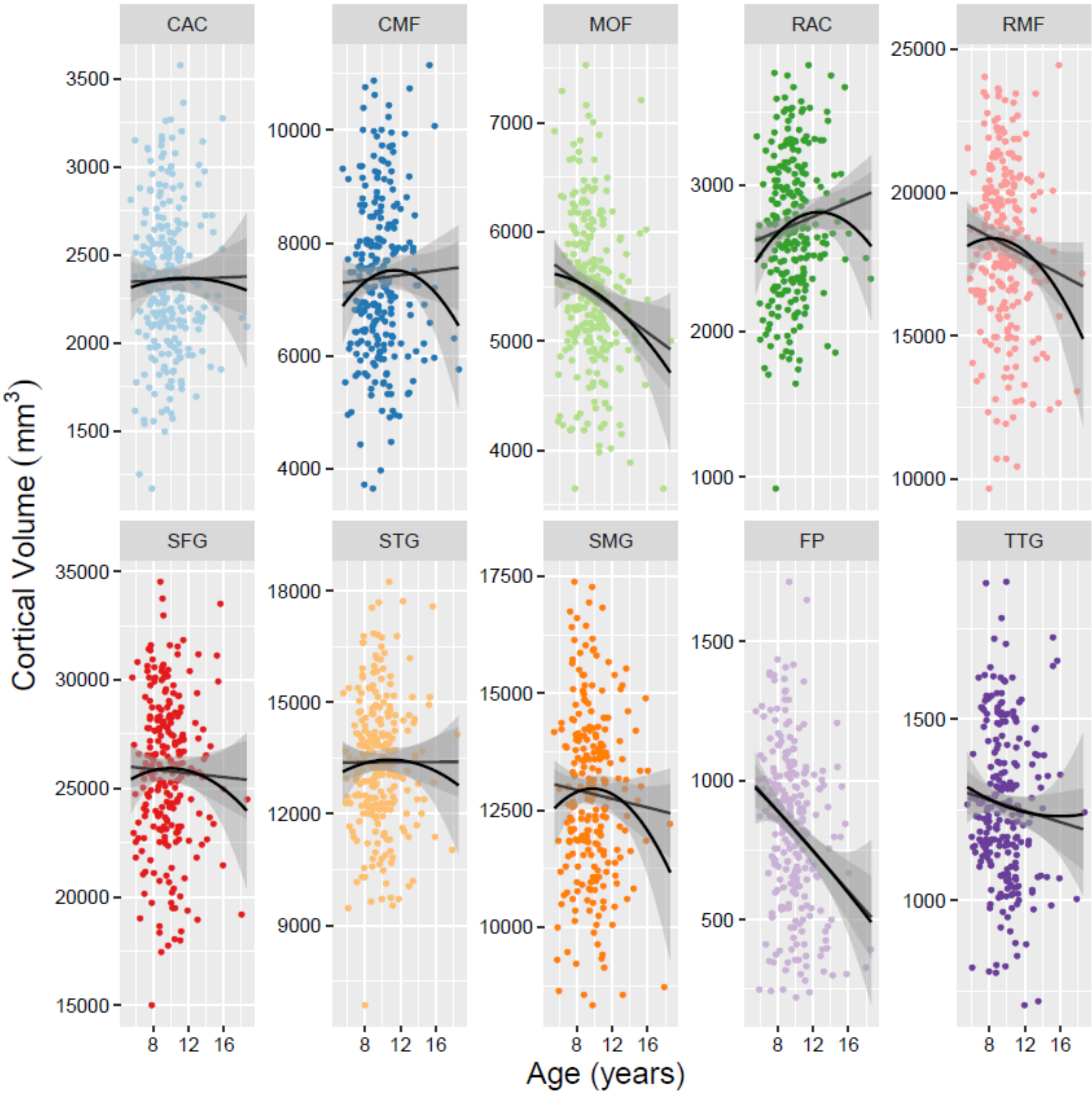
CAC
CMF
MOF
RAC
RMF
SFG
STG
SMG
FP
TTG
Cortical Volume (mm³)
Age (years)
8 12 16

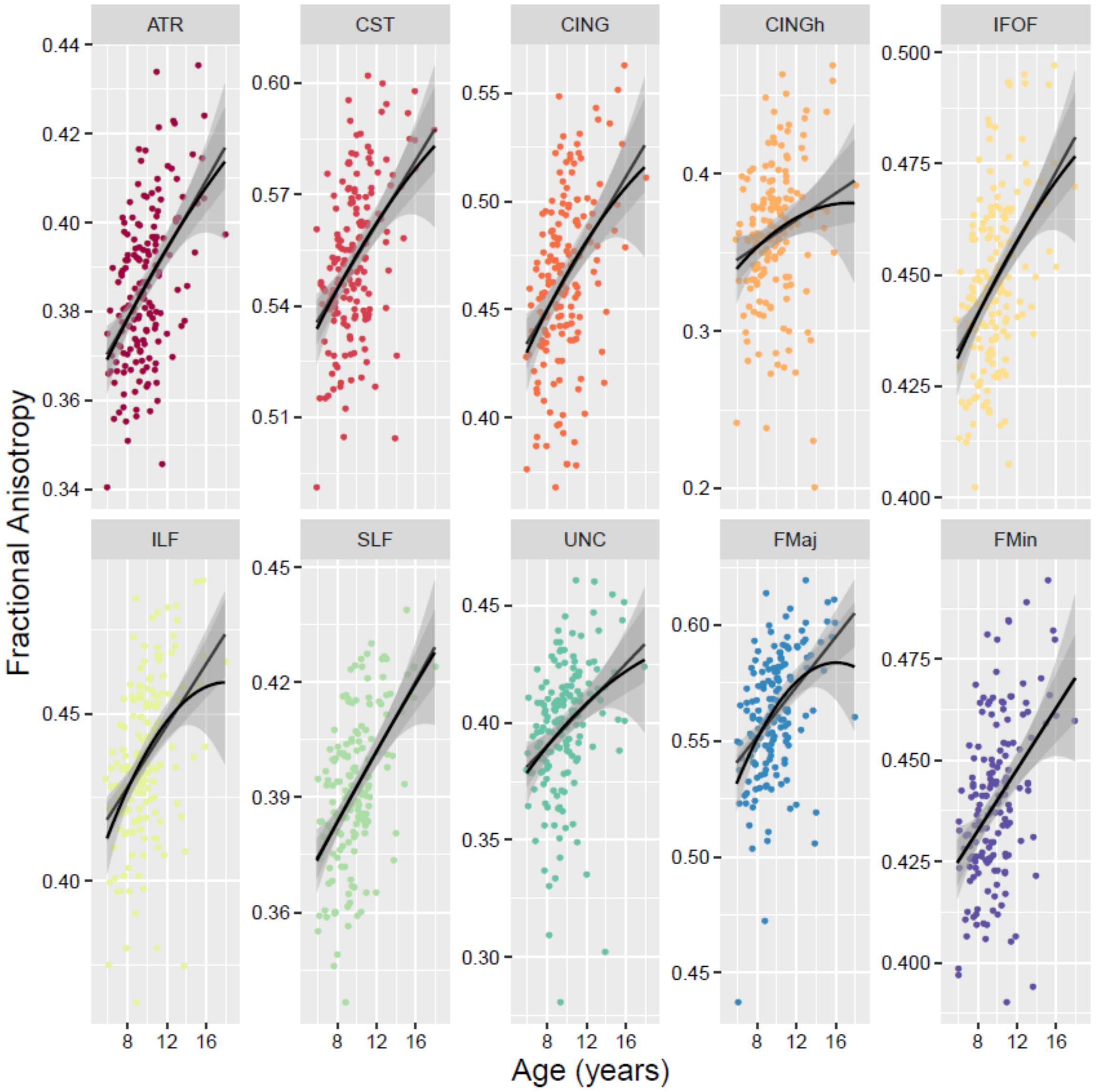

Supplementary Figure 1. Cross-sectional scatterplots for cognitive raw scores (top), bilateral cortical volume (middle), and bilateral fractional anisotropy (bottom). Solid lines represent linear and polynomial fit while shades indicate 95% confidence intervals. Abbreviations: matrix reasoning (MR), peabody picture vocabulary test (Pea), Spelling (Spell), single word reading (Read), numerical operations (NO), digit recall (DR), backward digit recall (BDR), Mr. X (MrX), dot matrix (Dot), following instructions (Ins), caudal anterior cingulate (CAC), caudal middle frontal gyrus (CMF), medial orbital frontal cortex (MOF), rostral anterior cingulate gyrus (RAC), rostral middle frontal gyrus (RMF), superior frontal gyrus (SFG), superior temporal gyrus (STG), supramarginal gyrus (SMG), frontal pole (FP), transverse temporal gyrus (TTG), anterior thalamic radiations (ATR), corticospinal tract (CST), cingulate gyrus (CING), cingulum [hippocampus] (CINGh), inferior fronto-occipital fasciculus (IFOF), inferior longitudinal fasciculus (ILF), superior longitudinal fasciculus (SLF), uncinate fasciculus (UNC), forceps major (FMaj), and forceps minor (FMin).

Edge-weight Stability Analyses

To further quantify the reliability of our partial correlation network edge-weights, we performed bootstraps (N = 2,000) and compared the bootstrapped mean values to the original sample estimates (Supplementary Figures 2-4). We do not show the bootstraps for the multilayer networks due to the size of the plots but they (and all code for this project) can be found online (https://osf.io/36d2n/). Bootstrapped edge-weight means were consistently near the original sample value with the most variable being the white matter network (Supplementary Figure 4) and the multilayer networks (not shown). The low edge-weight stability in these networks could possibly due to lower sample sizes of neural data (especially in the white matter network, N = 165, although centrality strength was moderately stable, CS-coefficient = 0.44), including when structural brain and cognitive data were combined. This, in turn, could have influenced the low stability estimates of the bridge centrality values in the multilayer networks.

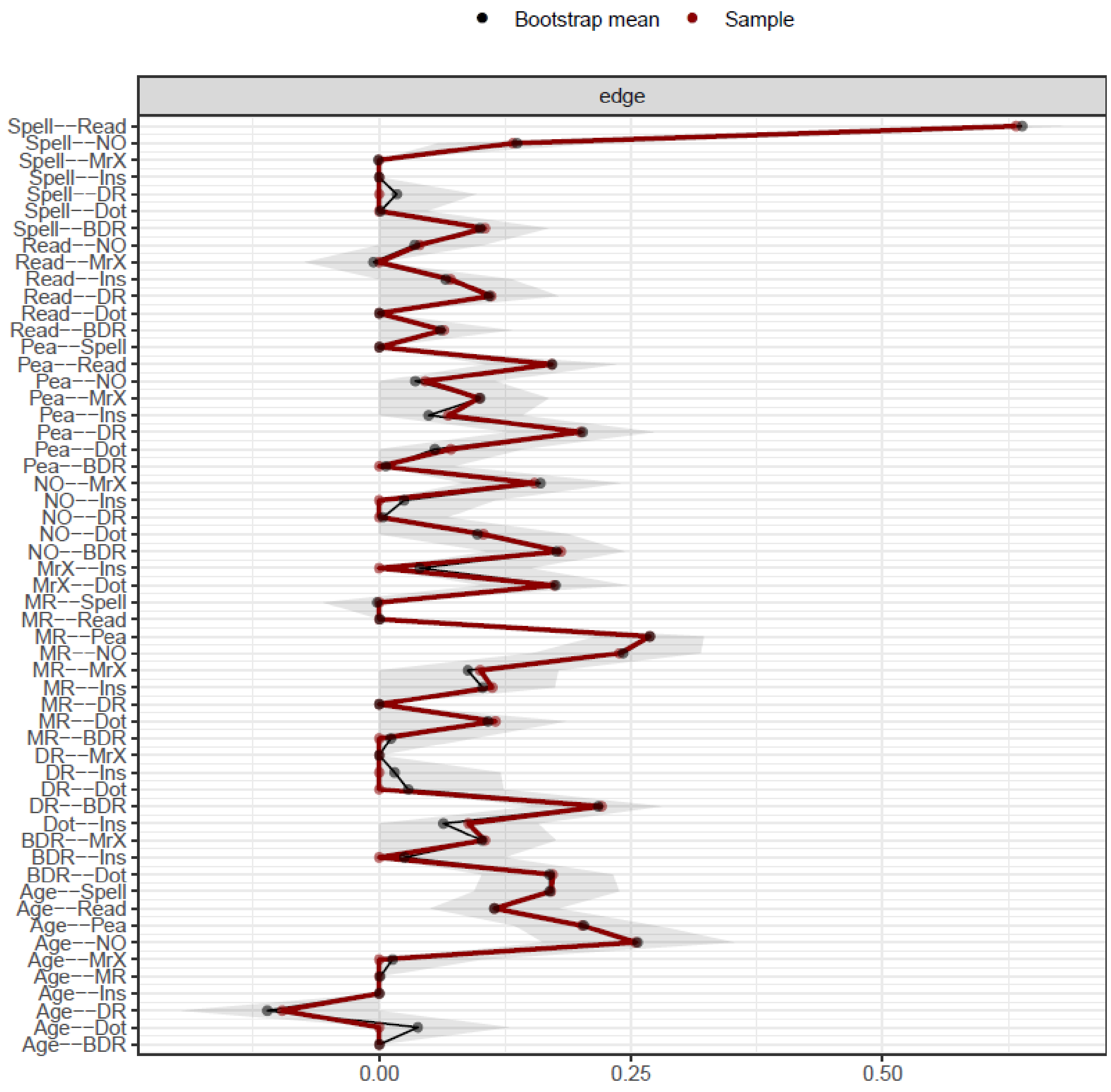

Supplementary Figure 2. Comparisons between bootstrapped means and original sample edge-weight estimates for the CALM cognitive partial correlation network.

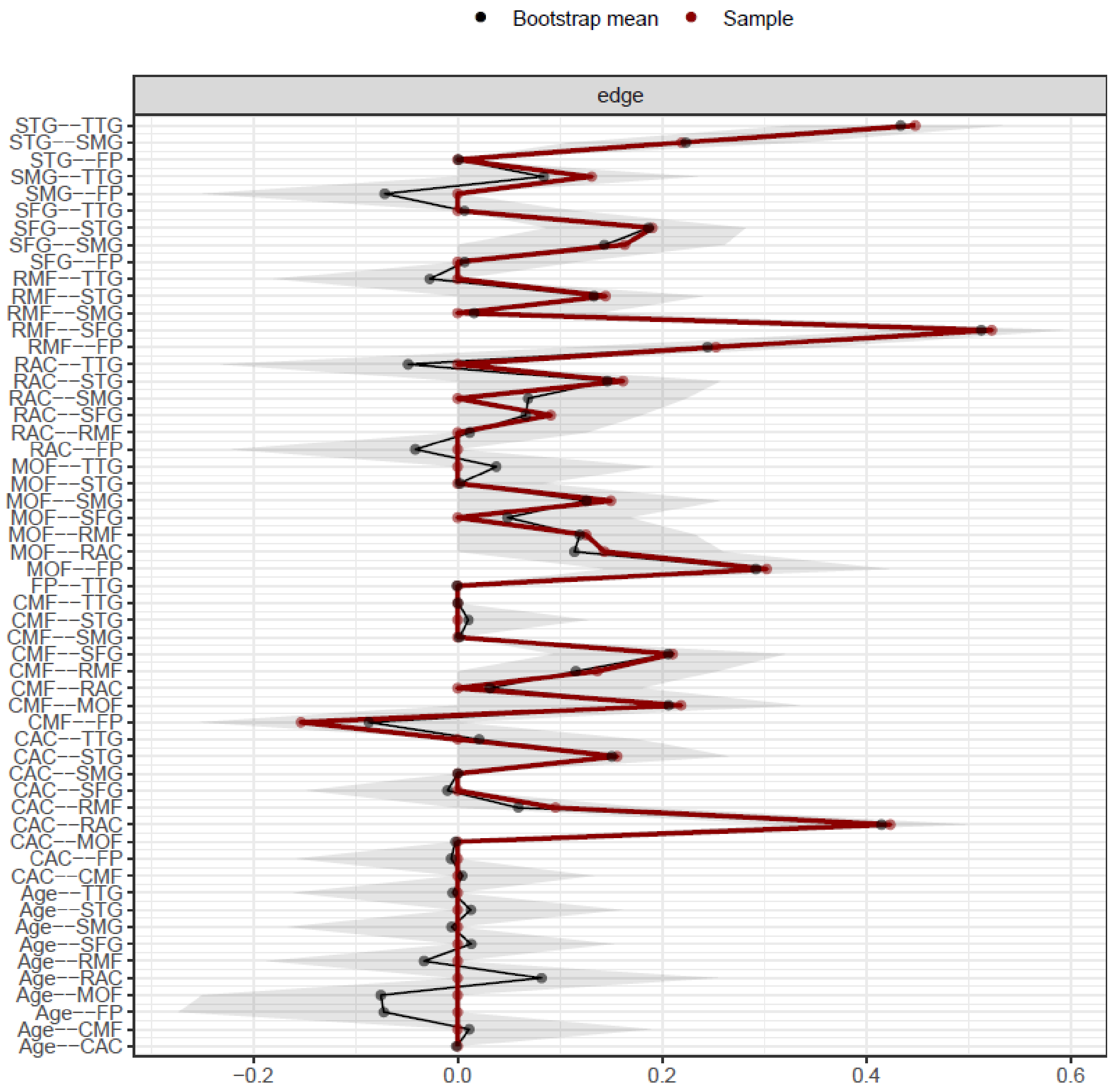

Supplementary Figure 3. Comparisons between bootstrapped means and original sample edge-weight estimates for the CALM grey matter partial correlation network.

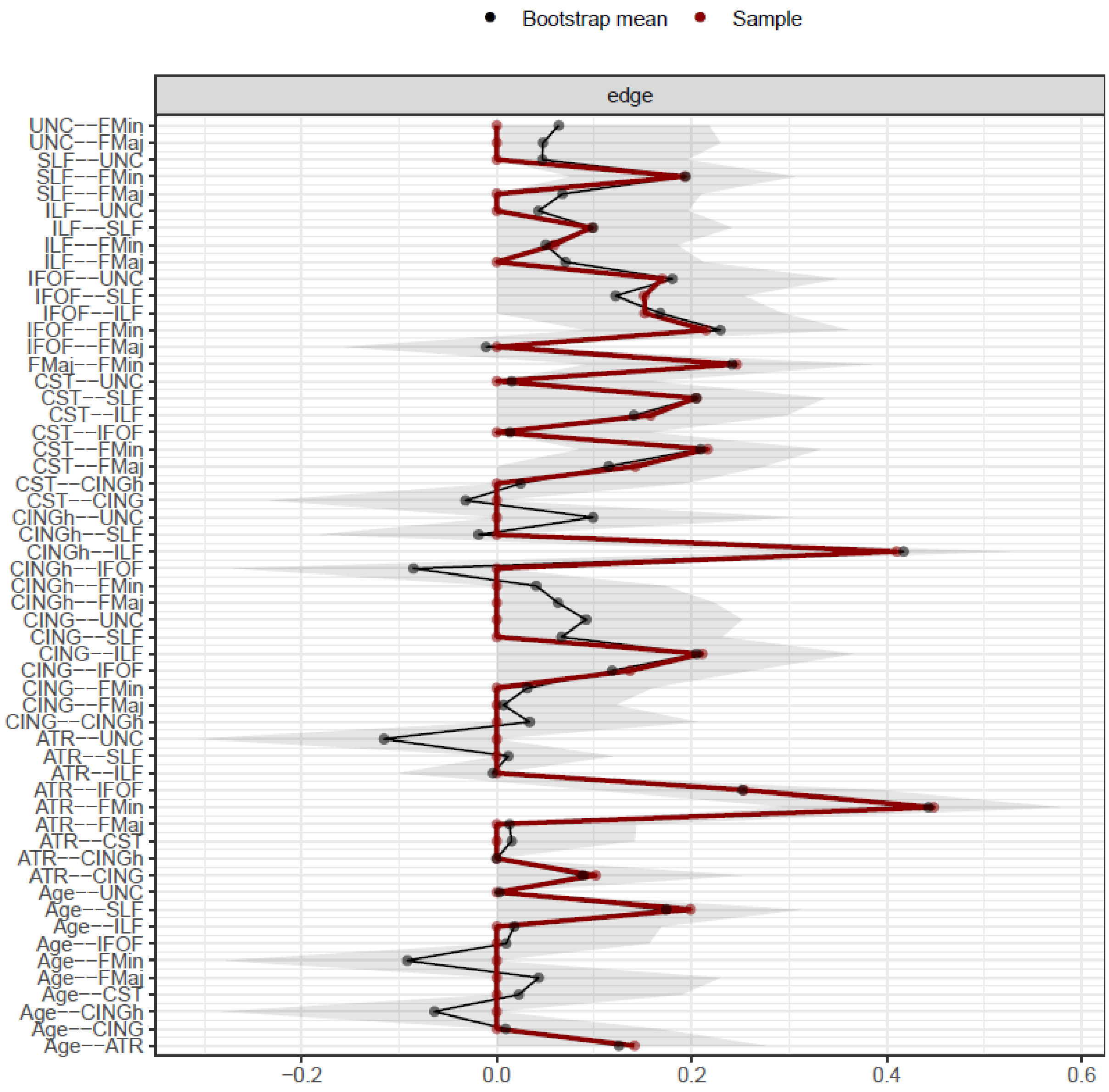

Supplementary Figure 4. Comparisons between bootstrapped means and original sample edge-weight estimates for the CALM white matter partial correlation network.

The Possible Effect of Outliers on Major Findings

In a previous version of this manuscript, we observed that two FA values (1 for the uncinate fasciculus, 1 for the forceps major), which represent potential outliers with undue influence on the partitioning of the Walktrap algorithm in the single-layer white matter network. Removing this data yielded a distinct, and more parsimonious clustering solution (2 communities vs. 5). Moreover, removing this outlier did not affect any summary statistics for the white matter partial correlation (single-layer) network except for range. Nevertheless, below we present the Pearson correlations between the weights obtained from the original data presented in the main manuscript and those from the data after all outliers (defined as ± 4 standard deviations) are removed (Supplementary Table 1). Due to the vast similarity in descriptive statistics and high correlations between partial correlation weights, we conclude that outliers did not confound the results of this study. However, it must be noted that outliers might slightly affect community detection, but we chose to keep the original data due to the nature of our sample (struggling learners, therefore behavioral and neural data might be atypical to begin with) and given the fact that the neural data was already quality controlled. Furthermore, the two outlier white matter ROIs occurred in two separate participants (1 outlier each) while the rest of their ROIs were consistent with the rest of the sample. In close, we argue that outliers (both cognitive and neural) are likely not due to measurement error but instead represent realistic values of an atypically developing sample.

Network Type	Original Data	Outliers Removed	Pearson Correlation
Cognitive	0.08 (0.11) [0, 0.63]	0.08 (0.11) [0, 0.61]	0.99
Grey Matter	0.09 (0.14) [-0.15, 0.52]	0.09 (0.14) [-0.15, 0.52]	1
White Matter	0.08 (0.11) [0, 0.44]	0.08 (0.13) [-0.14, 0.47]	0.93
Cognitive-grey matter	0.04 (0.1) [-0.12, 0.64]	0.03 (0.09) [-0.11, 0.62]	0.97
Cognitive-white matter	0.04 (0.1) [-0.2, 0.65]	0.04 (0.1) [-0.22, 0.65]	0.97
Tri-layer	0.02 (0.08) [-0.2, 0.66]	0.02 (0.08) [-0.19, 0.65]	0.98

Supplementary Table 1. Comparisons between partial correlation (PC) networks (original data vs. outliers removed). These include summary statistics such as mean, (standard deviation), [range], and Pearson correlations between PC graph weights using pairwise complete observations to account for missingness.

How to deal with age?

As in previous literature, in the CALM sample age shows a clear positive association with intelligence measures and brain structure (Supplementary Figure 1). This fact, however, may further complicate any interpretations of (possible) causal interactions between cognitive and/or neural nodes. This is due to the multitude of reasons age might correlate with cognition and brain structure. For instance, this pattern could be due to the fact that older participants normally score higher on cognitive tasks and have greater brain maturation. In this case age functions as an underlying driver of (even greater) covariance between the two domains. There are at least two options (included in the original preprint) of how to deal with the relationship of age to cognitive ability, and grey and white matter structural covariance: 1) We could estimate the partial correlation network and include age as a node, therefore, choosing to estimate it *simultaneously* with the cognitive and neural variables (this is the option we chose for the non-Supplemental part of the analyses), or 2) We could *regress out* the association of age for each variable (age would show no correlation with cognitive and/or neural measures) *before* network estimation. Both approaches are related and have corresponding pros and cons. For example, these two options might enable the detection of correlations beyond age, possibility revealing *core relations* among variables independent of stereotypical neurocognitive development (e.g., older participants normally score higher on cognitive tasks and have larger brains as they mature). However, this might also remove developmental associations of interest (e.g., age may function as a moderator of cognitive and neural growth as in the above example).

Notably, a third possible option, which addresses this limitation, is to estimate the network *ignoring* age (i.e., *removing* it from dataset *before* estimation). Specifically, choosing not to include age as a node has the benefit of revealing the 'actual correlations' (i.e., those dependent on neurocognitive development in childhood and adolescence) among cognitive abilities and brain structure in the population, as the 'effects' of age are not controlled for before (regressed out) or during (age node associations with other nodes removed during calculation of partial correlations) network estimation. However, a drawback to this approach is that doing so could also amplify these associations, confounding the findings.

Here we compare the partial correlations matrices for the three analysis paths (i.e., age node used in network estimation vs. age node regressed out before estimation; and age node used in network estimation vs. age node removed from dataset prior to network estimation) for both single and multilayer networks (Supplementary Tables 2 and 3). This analysis demonstrates that, regardless of how age is accounted for in estimation, the partial correlation networks are very similar to each other.

Network Type	Age Included in Estimation	Age Regressed Out before Estimation	Pearson Correlation
Cognitive	0.08 (0.11)<br>[0, 0.63]	0.08 (0.12)<br>[0, 0.65]	0.98
Grey Matter	0.09 (0.14)<br>[-0.15, 0.52]	0.09 (0.14)<br>[-0.15, 0.52]	1(rounded from 0.999)
White Matter	0.08 (0.11)<br>[0, 0.44]	0.08 (0.13)<br>[-0.2, 0.49]	0.93
Cognitive-grey matter	0.04 (0.10)<br>[-0.12, 0.64]	0.03 (0.10)<br>[-0.14, 0.65]	0.94
Cognitive-white matter	0.04 (0.10)<br>[-0.20, 0.65]	0.03 (0.10)<br>[-0.24, 0.66]	0.94
Tri-layer	0.02 (0.08)<br>[-0.20, 0.66]	0.02 (0.07)<br>[0, 0.64]	0.88

Supplementary Table 2. Comparisons between partial correlation networks (age included in estimation vs. age regressed out before estimation). These include summary statistics such as mean, (standard deviation), [range], and Pearson correlations between PC graph weights using pairwise complete observations to account for missingness.

Network Type	Age Included in Estimation	Age Removed from Dataset before Estimation	Pearson Correlation
Cognitive	0.08 (0.11)<br>[0, 0.63]	0.09 (0.12)<br>[0, 0.68]	0.99
Grey Matter	0.09 (0.14)<br>[-0.15, 0.52]	0.09 (0.14)<br>[-0.16, 0.52]	0.99
White Matter	0.08 (0.11)<br>[0, 0.44]	0.09 (0.13)<br>[-0.19, 0.46]	0.90
Cognitive-grey matter	0.04 (0.10)<br>[-0.12, 0.64]	0.04 (0.10)<br>[-0.11, 0.66]	0.97
Cognitive-white matter	0.04 (0.10)<br>[-0.20, 0.65]	0.04 (0.10)<br>[-0.21, 0.69]	0.97
Tri-layer	0.02 (0.08)<br>[-0.20, 0.66]	0.02 (0.08)<br>[-0.16, 0.67]	0.94

Supplementary Table 3. Comparisons between partial correlation networks (age included in estimation vs. age node removed from dataset prior to network estimation). These include summary statistics such as mean, (standard deviation), [range], and Pearson correlations between PC graph weights using pairwise complete observations to account for missingness.

Teasing Apart the Relations of Cortical Volume to General Intelligence: Multilayer Analysis Using Cortical Surface Area and Thickness

Lastly, we partitioned cortical volume into its constituent parts, cortical surface area and thickness, to compare their partial correlations and community structures when combined with white matter and general intelligence (Supplementary Figures 5 and 6). This produced bilayer networks that were much less connected between domains (brain vs. behavior) than the cognition-volume bilayer network in Figure 4 (top left). Finally, bridge strength showed the same pattern as in the main manuscript, except for the surface area tri-layer network, where neural regions (both grey and white) appear to dominate the bridge strength centrality (Supplementary Figure 6), rather than cognition (Figure 5, bottom).

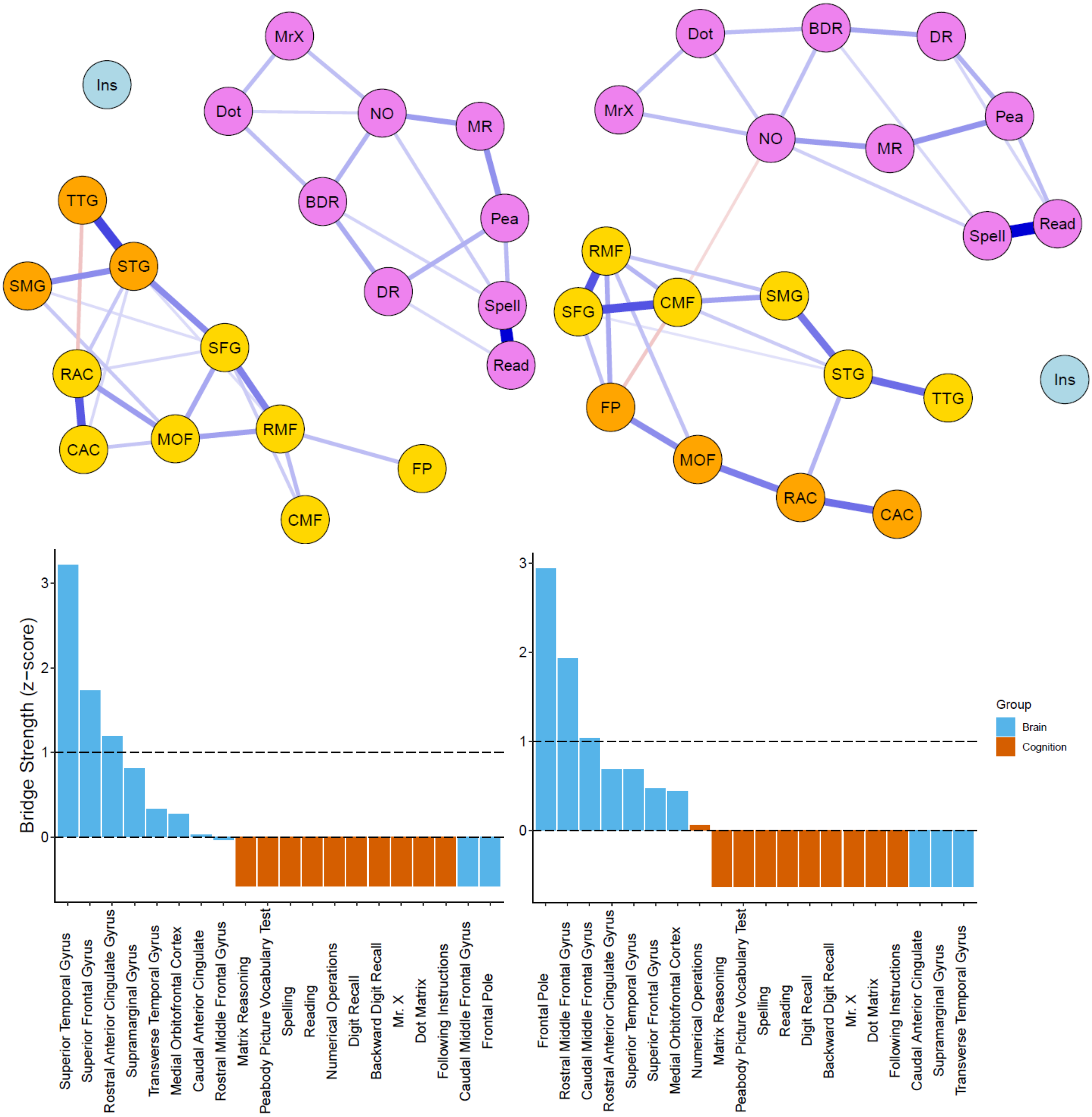

Supplementary Figure 5. Top: Network visualizations (spring layout) of partial correlation CALM bi-layer grey matter (surface area (left) and cortical thickness (right)) networks. Nodes are grouped according to Walktrap algorithm results. Bottom: Bridge centrality estimates (z-scores) for CALM bi-layer grey matter (surface area (left) and cortical thickness (right)) networks. Dashed lines indicate mean strength and one standard deviation above the mean.

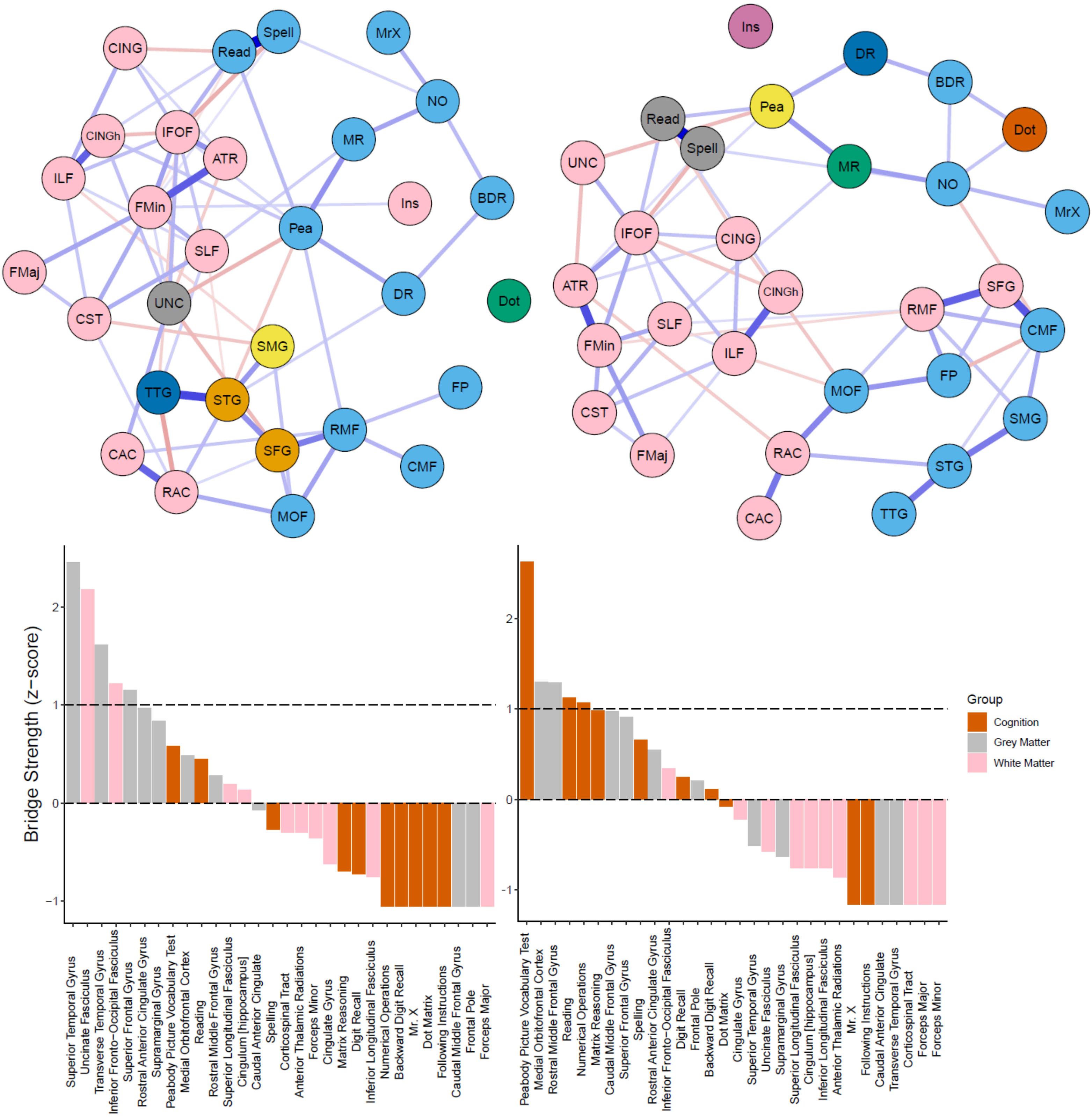

Supplementary Figure 6. Top: Network visualizations (spring layout) of partial correlation CALM tri-layer grey matter (surface area (left) and cortical thickness (right)) networks. Nodes are grouped according to Walktrap algorithm results. Bottom: Bridge centrality estimates (z-scores) for CALM tri-layer grey matter (surface area (left) and cortical thickness (right)) networks. Dashed lines indicate mean strength and one standard deviation above the mean.
